# Management and Treatment of Hepatitis C: Are There Still Unsolved Problems and Unique Populations?

**DOI:** 10.3390/v13061048

**Published:** 2021-06-01

**Authors:** Virginia Solitano, Maria Corina Plaz Torres, Nicola Pugliese, Alessio Aghemo

**Affiliations:** 1 Department of Biomedical Sciences, Humanitas University, Pieve Emanuele, 20082 Milan, Italy; virginia.solitano@humanitas.it (V.S.); nicola.pugliese@humanitas.it (N.P.); 2 Division of Internal Medicine and Hepatology, Humanitas Research Hospital IRCCS, Rozzano, 20089 Milan, Italy; maria.plaztorres@humanitas.it; 3 Gastroenterology Unit, Department of Internal Medicine, University of Genoa, IRCCS-Ospedale Policlinico San Martino, 16132 Genoa, Italy

**Keywords:** hepatitis C, unique populations, special populations, decompensated cirrhosis, chronic kidney disease, end stage renal disease, HIV/HBV coinfection, rare genotypes, treatment failure

## Abstract

Direct-acting antivirals (DAA) have revolutionized the treatment of patients with chronic hepatitis C virus (HCV) infection, possibly leading to HCV elimination by 2030 as endorsed by the World Health Organization (WHO). However, some patients belonging to the so-called unique or special populations are referred to as difficult-to-treat due to unreached sustained virological response, potential drug side effects or interactions or co-morbidities. Several years after the DAA introduction and on the basis of excellent findings in terms of efficacy and safety, some doubts arise around the exact meaning of the special population designation and whether this group of patients actually exists. The aim of this review is to discuss and analyze current evidence on the management and treatment of the so-called “unique populations”. We placed particular emphasis on patients with decompensated cirrhosis, chronic kidney disease (CKD), coinfections, rare genotypes, and previous treatment failure, in order to provide physicians with an updated overview of the actual problems and needs in the current scenario.

## 1. Introduction

Direct-acting antivirals (DAA), including RNA-dependent polymerase inhibitors (anti-NS5B), protease inhibitors (PI, anti-NS3/4A) and anti-NS5A inhibitors, have radically changed the landscape of patients with chronic hepatitis C virus (HCV) infection [1]. The availability of DAA together with the improvement of worldwide access to treatment could potentially lead to HCV elimination by 2030 as endorsed by the World Health Organization (WHO) [2]. However, some patients have historically been part of the so-called unique or special populations [3]. This term generally refers to those who are difficult-to-treat due to unreached sustained virological response (SVR), potential treatment side effects or drug interactions or co-morbidities [4].

In this context, the presence of liver decompensation and severe renal impairment may limit treatment options [5]. Moreover, due to the risk of re-activation of hepatitis B virus (HBV) in patients with dual infections and to the risk of drug interactions in human immunodeficiency virus (HIV) co-infected individuals, careful consideration before therapy initiation is needed [6,7]. Notably, less treatment-susceptible genotypes (e.g., 1l, 4r, 3b, 3g, 6u, 6v) is an emerging challenge and DAA treatment failure involves a significant proportion of patients (approximately 1–3% of all HCV patients) [8,9].

Several years after the DAA introduction, some doubts arise regarding the exact meaning of the special population designation and who should belong to this group [10].

In this review, we aim to collect current evidence on the management and treatment of special populations that have been traditionally considered difficult-to-treat due to pharmacological, virological, and co-morbidity reasons. In particular, the review focuses on patients with decompensated cirrhosis, chronic kidney disease (CKD), coinfections, rare genotypes and previous treatment failure, in order to provide with an updated overview of the unsolved problems and needs in the 2021 scenario.

## 2. Patients with Decompensated Cirrhosis

**Figure 1:**
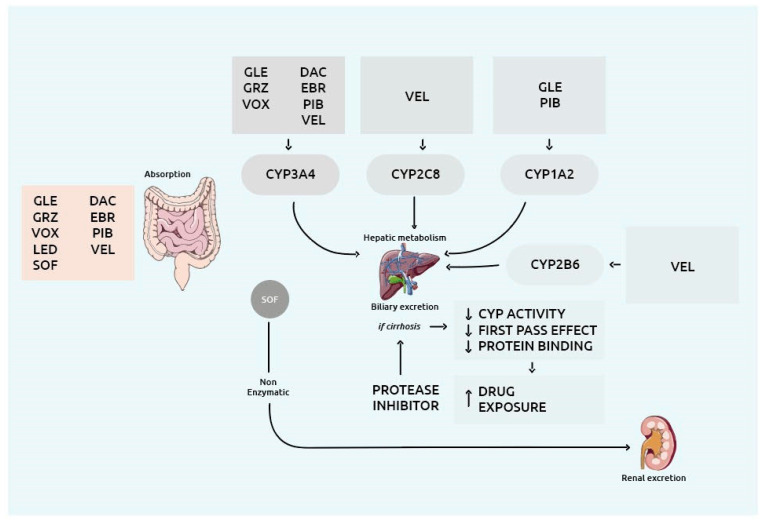
Overview of the drug metabolism enzymes and drug transporters involved in the metabolism and distribution of the several DAA. *Abbreviations:* CYP: cytochrome P450; DAC: daclatasvir; EBR: elbasvir; GLE: glecaprevir; GRZ: grazoprevir; LED: ledipasvir; PIB: pibrentasvir; SOF: sofosbuvir; VEL: velpatasvir; VOX: voxilaprevir.

Patients with decompensated (Child-Pugh B or C) HCV-related cirrhosis have always been considered a difficult-to-treat cohort for which treatment options were limited and only liver transplantation could represent a definitive cure [11]. Due to the substantially elevated drug exposure and liver injury associated with NS3/4A PI, PI-containing regimens are not allowed in HCV patients with current or past decompensated cirrhosis [12]. Occurrence of liver failure or death due to the use of NS3/4A PI has been reported, and in 2019 the Food and Drug Administration (FDA) raised a safety warning about PI-containing regimens in these patients [13]. Figure 1 summarizes metabolism and distribution of the several available DAA.

Among non-PI based DAA, the treatment of choice for patients with current decompensated cirrhosis or with compensated (Child-Pugh A) cirrhosis with prior episodes of decompensation consists of the fixed-dose combination of sofosbuvir (SOF) and velpatasvir (VEL) regimens. The paradigmatic ASTRAL-4 phase III randomized trial evaluated the efficacy and safety of a fixed-dose combination of SOF/VEL with or without ribavirin (RBV) for 12 weeks or SOF/VEL for 24 weeks in 267 treatment-naïve and treatment-experienced decompensated patients (Child-Pugh class B) [14]. Notably, overall rates of SVR at 12 weeks after the end of treatment (SVR12) were 83% in patients treated without RBV (including all genotypes), compared to 94% among those receiving SOF/VEL plus RBV for 12 weeks [14]. However, considering only patients with HCV genotype 3 (15%), the SVR was 85% in those receiving SOF/VEL plus ribavirin, dropping to 50% in those receiving the same DAA combination without ribavirin [14].

Data from the real-world multicenter TARGET study, including 170 decompensated patients showed that treatment with ledipasvir (LDV)/SOF with or without RBV led to optimal response rates (SVR12 88%, regardless of HCV genotype) [15]. Consistently, other two real-world studies from the United Kingdom (UK) including large cohorts of patients with decompensated cirrhosis showed that anti-HCV DAA treatment is beneficial even in this group of patients [16,17]. A more recent Japanese real-world study reported an SVR12 rate of 90% in a cohort of 82 patients with decompensated cirrhosis and significant improvements in Child-Pugh score after treatment. For instance, 50% of patients with class B at baseline reached class A Child-Pugh score whereas around one third and one tenth of baseline class C Child-Pugh patients were class B and A, respectively, at the end of treatment [18].

Considerably, the cost-effectiveness of SOF/VEL plus RBV in advanced decompensated patients has been recently highlighted by a state-transition model that compared this DAA combination therapy with best supportive care [19]. SOF/VEL decreased the range of liver-related deaths and progression to Child Pugh C cirrhosis by 20.4% and 21.9%, respectively, with an incremental cost-effectiveness ratio of $39,169 per quality-adjusted life years [19].

Despite clinical trials and real-world studies showing excellent rates of SVR and cost-effectiveness with SOF-based regimens with a lower decompensated cirrhosis risk [20], predictors of response in this peculiar group are scarce. Interestingly, Debnath and co-workers (2020) found that albumin, alanine transaminase (ALT), bilirubin, and estimated glomerular filtration rates (eGFR) were independently associated with treatment response (odds ratio [OR] = 4.84, 1.02, 0.41, 1.03 for the above-mentioned variables, respectively; *p* < 0.05) [21]. In addition, patients with a model for end-stage liver disease (MELD) score >15 and a Child Pugh C score did not achieve a persistent compensated state 36 weeks after treatment (primary end-point) and authors concluded that early liver transplantation should be mandatory in this case [21].

Not to be overlooked, a recent long-term (median follow-up of 4 years) evaluation of patients with advanced or decompensated cirrhosis (MELD ≥ 10) showed marginal (0.3 point) and not-meaningful improvement in MELD score after an NS5A-containing DAA treatment (reduction of MELD by 3 or more points and MELD score of <10 in 25% and 29%, respectively), regardless of SVR [22]. Therefore, the impact of SVR response on liver function should not be optimistically overestimated in this group of patients that still require close monitoring and special attention [22].

## 3. Patients with Renal Impairment, Including End-Stage Renal Disease on Hemodialysis

The reciprocal relationship between HCV infection and CKD is well established [23]. Robust data demonstrated that chronic HCV infection can cause per se renal impairment which may be secondary to HCV-related mixed cryoglobulinemia or, in the absence of cryoglobulinemia, to direct viral invasion of the renal parenchyma, and to nephrotoxicity of drugs used for its treatment [23,24]. These mechanisms often interact in the pathogenesis of several acute and chronic clinical renal syndromes [23,25]. The histopathological lesions vary from segmental glomerulosclerosis to membrane-proliferative glomerulonephritis and interstitial nephritis [26]. On the other hand, especially in countries where HCV is highly prevalent (such as Russia, Egypt, Syria, Georgia, and others in southern and eastern Asia), HCV is frequently detected in patients with a pre-existent renal impairment due to other clinical conditions [27].

Moreover, end-stage renal disease requiring dialysis may increase the risk of HCV-infection, especially in dialysis units where hygienic measures are poor [23,24]. HCV-infection, in turn, raises the risk of mortality in dialysis population and that of hepatic function deterioration in patients receiving kidney transplantation [23,24].

In this scenario, anti-viral treatment with DAA may play a pivotal role in the amelioration and even prevention of renal impairment [25]. For instance, El-Serag et al. (2019) showed that successful HCV treatment with DAA actually prevented the onset of glomerulonephritis in a population of 45,260 male subjects from the US Department of Veterans Affairs Corporate Data Warehouse [28]. In the treated cohort the risk of CKD was significantly reduced as compared with the untreated group (adjusted hazard ratio [aHR] = 0.61; 95% confidence interval [CI], 0.41–0.90). Additionally, remissions from glomerular disease have been reported in patients achieving SVR after treatment with DAA, although data on the improvement of renal lesions after successful DAA treatment are scarce [29]. However, organ recovery may be delayed after an SVR in patients with cryoglobulinemia [30].

Whilst no safety concerns are present for CKD patients undergoing DAA treatment with either the combination of glecaprevir (GLE)/pibrentasvir (PIB) or elbasvir/grazoprevir [25,31] independently from the disease stage and including those on hemodialysis, renal safety of the treatment combinations based on SOF has been questioned [32]. Indeed, data regarding the safety of SOF in renal impairment are controversial [33,34,35,36].

In 2016 Saxena et al. reported a higher frequency of renal function worsening and serious adverse events (AEs) (all *p* < 0.05) in patients with a baseline eGFR < 45 mL/min treated with SOF-containing regimens [36]. However, a significant number of studies aimed at verifying the safety of SOF-based regimens in patients with an established diagnosis of CKD, showed no significant changes in renal function [33] nor AEs associated with renal function, even in patients receiving dialysis [34,35]. The safety of the SOF/VEL combination has been recently evaluated in a 12-week non-controlled study including 59 patients with end-stage renal disease requiring hemodialysis [34]. The exposure of SOF metabolite GS-331007 was increased 20-fold, exceeding levels where adverse reactions have been observed in preclinical trials. This notwithstanding, the rate of AEs and deaths was not higher than expected in patients with end stage renal disease [34].

Available data consistently showed optimal rates of SVR with all the approved pan-genotypic treatments in patients with severe CKD and on hemodialysis, including full-dose SOF-based regimens [33,34,35]. The magnitude of recent studies confirming the safety profile of SOF is of fundamental importance in contexts where resources are limited and in cases of decompensated cirrhosis where SOF-based regimens with or without RBV represent the only option [33,34,35,37]. As a result, patients with HCV infection and concomitant CKD do not appear as a difficult-to-treat population to date and IFN-free pan-genotypic treatments can be recommended in all patients with kidney disease, without dose-adjustments [25,38].

## 4. Patients with HBV/HIV Coinfections

### 4.1. Patients with Concomitant HBV Infection

In patients with HCV and HBV coinfection, DAA treatment should be prescribed similarly to HCV-mono-infected patients. However, HBV re-activation in HBsAg-positive patients may occur during and after DAA treatment in a considerable proportion of patients [39,40,41]. The underlying mechanisms of HBV re-activation during/after pan-oral DAA treatment may rely on the HCV-related suppression of HBV replication but the exact pathway of such suppression is poorly understood [39].

Cases of HBV re-activation (detected by the evidence of quantifiable HBV-DNA levels or an increase >1 log10 IU/mL from baseline) and ALT elevations > 2 times upper normal level have been reported in up two thirds of HBsAg positive patients treated with DAA [40,42,43]. In most patients the increase of HBV DNA levels was not associated with signs or symptoms of hepatitis and ALT elevations were mostly mild [40,43,44,45]. Still, approximately 4% of patients with HBV re-activation after DAA treatment need to start antiviral treatment [39,40] and importantly, HBV re-activation in cirrhotic patients may lead to liver failure and death despite immediate nucleoside or nucleotide analogue (NUC) therapy [46].

As such, taking into account that no strong predictive factors for HBV re-activation have been demonstrated, the most recent European Association for the Study of the Liver (EASL) guidelines recommend to start concurrent prophylactic HBV NUC therapy in all HBsAg-positive patients commencing DAA-based treatment [25]. Differently from EASL recommendations, the American Association for the Study of Liver Disease (AASLD) states that monitoring HBV DNA and ALT every 4–8 weeks for 3 months post-DAA is appropriate in co-infected patients positive for HBsAg [47]. The lack of unanimous agreement regarding the management of these patients suggests that HCV/HBV co-infected are still a special population and future studies are expected to clarify the best therapeutic approach for this cohort [6].

### 4.2. Patients with HIV Co-Infection

HCV patients co-infected with HIV carry a higher risk of fibrosis progression leading to a faster occurrence of cirrhosis and related complications [48]. HIV infection, indeed, causes direct and indirect liver injury due to interactions with hepatic stellate cells and the immune system which accelerate extracellular matrix production and pro-fibrotic pathways [49]. Moreover, HCV/HIV co-infected patients show higher rates of decompensation and HCV-viral loads, with an increased risk of drug interactions and altered absorption of antivirals [50,51].

Since the advent of DAA, several trials have been conducted addressing the outcomes for the unique population of HCV/HIV co-infected patients. All studies reported optimal efficacy and safety results. In particular SVR rates for patients with dual HCV/HIV infection treated with pan-genotypic regimens were comparable to those with HCV mono-infection [52,53,54,55,56,57,58,59,60,61,62,63].

For instance, in the ASTRAL study, including 106 patients treated with SOF/VEL, SVR rate was 95% (95% CI: 89–99%). In the EXPEDITION-2 and ENDURANCE-1 studies, which investigated the safety and efficacy of GLE/PIB in HIV/HCV co-infected patients SVR rates ranged from 95% for HCV-3 to 99% for HCV-1 infected patients, respectively. These SVR rates are comparable to those of the HCV mono-infected population [54,55].

In summary, DAA pan-genotypic treatments can be safely recommended in HIV/HCV co-infected subjects [64]. Indeed, the most recent European AIDS Clinical Society (EACS) guidelines recommended timely HCV therapy in HIV-positive patients with low CD4-count (<200/µL), with persistent HIV-viremia and HBV-coinfection [65]. No drug interactions nor lower SVR rates are expected in HIV-infected patients treated with DAA based on the combination of GLE/PIB or SOF/VEL or SOF/VEL/voxilaprevir (VOX) [65,66]. Attention is required only when LDV-based regimen is prescribed in patients treated with tenofovir and ritonavir-boosted HIV protease-inhibitors [64]. In addition, LDV/SOF treatment should not be prescribed in patients treated with cobicistat, elvitegravir and tipranavir [64]. The latest AASLD guidelines suggested the collaboration between hepatologists and infectious disease specialists when drug switches are needed [64]. However, given the paucity of drug-drug interactions and the safety of DAA, such cases are rare and most patients continue antiretroviral treatment during HCV treatment safely [64].

In conclusion, though antiretroviral HIV drugs should be taken into account before starting DAA treatment and physicians should always consider possible barriers impacting SVR such as drug injection, alcohol abuse, psychiatric disorders, and incarceration, it seems inappropriate to still regard HCV/HIV patients as special populations [67].

## 5. Patients with less Treatment-Susceptible HCV-Subtypes

While the WHO set the objective to eliminate hepatitis C by 2030, only few wealthy states seem able to reach the goal, among a vast majority of low-middle income countries [68,69]. As one of the causes for the unlikely HCV elimination in the planned timeframe, it should be mentioned that DAA combinations may not be as pan-genotypic as claimed [70]. According to the EASL guidelines, pan-genotypic antiviral regimens, including GLE/PIB and SOF/VEL, can be administered without the need to identify HCV genotype and subtype. However, based on the most recent literature, we think that genotype and subtype identification before starting the treatment appears useful in specific settings (such as in patients from geographical areas where less treatment-susceptible subtypes are known to be prevalent) in order to optimize treatment regimens [5].

Indeed, some subtypes of genotypes 1 to 8 that are uncommon in Western countries have been shown to be prevalent in some regions of Asia and Africa and among migrants from these areas (defined as genotype 1 non-1a/1b, genotype 2 non-2a/2b, genotype 3 non-3a, genotype 4 non-4a/4d, and subtypes of genotypes 5 to 8) [71,72,73]. Data on treatment outcomes in these uncommon genotypes and subtypes are limited.

In a large-scale single arm trial performed in Rwanda, Gupta et al. (2019) showed that genotype 4r, found in 16% of patients, was associated with high rates of treatment failure with LDV/SOF in combination (SVR12 = 56% vs. 93%, respectively), despite appropriate compliance [74]. This could be associated to the existence of an amino acid motif of the NS5A protein that confers high-level resistance to DAA in vitro [75]. In accordance with these findings, the French National Reference Centre recently described that subtype 4r is associated with lower SVR rates [76]. Among DAA-treated patients who experienced a virological failure in France between 2015 and 2018, 22.5% were infected with genotype 4, more specifically 5% with subtype 4r, which is rare among the French population. Treatment failure was related to multiple NS5A resistance-associated substitutions (RASs) which were actually present in all subtype 4r patients at baseline [76].

Consistently with the previous studies, Childs et al. (2019) reported suboptimal rates of SVR in a London cohort of African patients infected with uncommon HCV genotype subtypes [71]. Over an eight-year period, the authors identified 91 African-born patients, 35 (38.5%) of those infected with unusual HCV genotype 1 subtypes (including 1e, 1g, 1h, 1l or unassigned genotype 1) and 12 (13.1%) with unusual genotype 4 subtypes (including 4c, 4e, 4f, 4k and 4r). After DAA treatment (SOF/LDV, paritaprevir/ritonavir/ombitasvir dasabuvir, or elbasvir/grazoprevir), SVR was observed only in 75% African patients infected with uncommon genotype 1 subtypes (1p, 1l) compared to 100% in all other genotypes and subtypes. In this context, as well as in the above-mentioned studies, failures of NS5A inhibitor-containing regimens, were explained by the frequency of NS5A RASs that could be considered as a natural polymorphism in African subtypes [71].

On the contrary, reassuring data come from a study by Zeuzem et al. (2017)., including 25 participants with uncommon genotype 1 subtypes, such as 1c/e/g/h/l. All subjects achieved SVR with an NS5A inhibitor-based regimen (LDV/SOF, SOF/VEL, or SOF/VEL/VOX) [77]. However, it should be mentioned that most patients received either LDV/SOF for 24 weeks or SOF/VEL (with or without VOX). Therefore, it is plausible that such a successful virological response, despite high rates of RASs, was influenced by either the use of the most potent antiviral regimen available or the prolonged treatment [77].

In view of the above, people from areas with a high prevalence of less treatment-susceptible HCV subtypes, may benefit from determination of genotype and subtype or deep sequencing of the NS5B so as to avoid treatment failure. Moreover, clinical trials and real-world studies are necessary to define the efficacy of DAA regimens against such unusual subtypes.

Therefore, it can be affirmed that patients with unusual genotypes (such as 1l, 4r, 3b, 3g, 6u and 6v) represent a unique population and that assessing the efficacy of DAA treatment in these constitutes a priority challenge in the near future in order to obtain HCV elimination worldwide. In our opinion, due to the scarcity of data, patients infected with uncommon subtypes should be treated with 12 weeks of the fixed combination of SOF/VEL/VOX [5].

## 6. Patients with Previous DAA Failure

While failure to DAA is rare, SVR rates have reached 95–99% across all HCV genotypes, the cohort of HCV patients requiring retreatment with DAA-salvage treatments is numerically large [78,79].

Factors associated with DAA failure include decompensated cirrhosis, lack of compliance, presence of hepatocellular carcinoma, and uncommon HCV genotype subtypes [80,81]. DAA treatment failures also occur in instances characterized by high prevalence of RASs in the region targeted by the administered drugs. Of note, NS5A RASs prior to therapy had a negative impact on virological response, leading to lower SVR rates (76% vs. 97% among treatment-experienced genotype 1a patients) [82]. On the contrary, NS5B protein’s RASs limiting the efficacy of drugs such as SOF are harder to detect, as these variants typically replicate very poorly [83].

Many guidelines recommend RASs testing prior to re-treatment in patients who do not achieve SVR, but the benefit and cost-effectiveness of this approach has not been consistently documented [5,84].

As a matter of principle, re-treatment should be based on SOF-based regimens, as a drug with the highest genetic barrier to resistance [9]. In addition, regimens containing the same class of DAA that caused the failure should be avoided [9]. Finally, if resistance testing is performed, it is worth considering probabilities of response according to the resistance profile observed [9].

According to the most recent EASL guidelines, the first line treatment for DAA-failure is a 12 weeks SOF/VEL/VOX regimen. The evidence for such recommendation was initially derived from the POLARIS 1 and 4 trials [85]. In the POLARIS-1 study, patients (genotype 1−6) who failed an NS5A inhibitor-containing regimen achieved a 96% SVR12 (253/263) following a 12-week course of SOF/VEL/VOX. The POLARIS 4 trial confirmed the superiority of this triple therapy versus a double drug regimen (SOF/VEL) in genotype 1-4-infected patients with prior NS5B-inhibitor and/or NS3-PI (but not NS5A-inhibitor) treatment failure [85]. Subsequently, several real-world studies confirmed the high efficacy and safety of the triple combination regardless of patient gender, HCV genotype, and baseline HCV RNA [81,86,87]. The only pre-treatment parameter associated with a lower SVR rate in SOF/VEL/VOX treated patients was the presence of advanced liver disease (F4) [81].

A valid alternative to SOF/VEL/VOX for patients with predictors of poor response, such as advanced cirrhosis, complex RASs patterns, or failure to multiple courses of treatment, is a 12-week course of SOF+GLE/PIB [5]. In fact, a phase IIIb trial showed that 22 out of 23 patients who were retreated with the triple combination of SOF+GLE/PIB for 12 or 16 weeks after a GLE/PIB-regimen failure achieved SVR12 (96%) [88].

A “very difficult-to-treat” population is represented by subjects with NS5A RASs who failed twice to achieve an SVR after a combination regimen including a PI and/or an NS5A inhibitor. The management of these patients has not been yet resolved. The EASL guidelines suggest the possibility of using the above-mentioned triple regimens (SOF/VEL/VOX and SOF + GLE/PIB) with the addition of RBV [5]. However, this recommendation remains to be validated by prospective studies in the near future.

Patients with decompensated cirrhosis and prior DAA failure are another “very difficult-to-treat” population, as PI are contraindicated, and the only recommended regimen is SOF/VEL combination therapy plus RBV for 24 weeks. Nevertheless, given possible baseline NS5A RASs and poor tolerance to full course RBV, this combination is associated with suboptimal SVR rates, especially in HCV-3 patients (78% [95% CI: 52–94%] in HCV-3 vs. 97% [95% CI: 86–100%] and 93% [95% CI: 66–100%] in HCV-1 and HCV-2, respectively) [89].

In summary, the available evidence highlights that patients with previous DAA failures with or without negative prognostic factors (e.g., decompensated cirrhosis) constitute a difficult-to-treat population and that further studies providing consistent data on the best therapeutical approach for these patients are necessary [9].

## 7. Conclusions

DAA have undoubtedly allowed all HCV-infected patients to be potentially cured of their disease. Moreover, in most patients belonging to groups historically considered difficult-to-treat (e.g., CKD, HCV/HIV co-infected) the SVR rates are optimal with very good safety and tolerability profiles (Table 1).

Patients with CKD or needing hemodialysis do not require special attention, and the use of SOF-based regimens in these cases has been endorsed by the latest EASL guidelines. Moreover, subjects with HCV/HIV co-infection are no longer to be considered a unique population and therefore attention should be paid only to the few interactions between DAA and antiretroviral HIV drugs.

However, unsolved problems persist even in the era of pan-genotypic DAA. Despite achieving SVR in most cases, patients with decompensated advanced liver disease remain exposed to long-term complications (such as hepatocellular carcinoma, ascites, hepatic encephalopathy, variceal bleeding) and HCV elimination might lead these subjects to be listed at a lower priority for liver transplantation [90]. As a result, we believe this group still requires close monitoring and special attention.

Furthermore, given the clinically relevant risk of reactivation of HBV after DAA cure, uncertainties about the best therapeutic approach for patients with dual HCV/HBV infection are still present and the lack of agreement among international societies’ guidelines makes the issue controversial.

In addition, the management of patients with unusual subtypes (e.g., 1l, 4r, 3b, 3g, 6u and 6v) and of those with prior DAA treatment failure is still a matter of concern. Furthermore, the co-existence of negative prognostic factors such as decompensated cirrhosis makes these subjects even more difficult-to-treat; hence, future studies are expected to clarify the best DAA regimen to administer to these cohorts.

## Figures and Tables

**Table 1:** Unique populations over time.

Population	Past	Current State of the Art	Future Perspectives
Decompensated cirrhosis	Unique population due to higher risk for decompensation and side effects during antiviral therapy	Special attention required due to exposure to long-term complications. Negative prognostic factor overall	Not overestimate the impact of SVR on liver function and not overlook the possible persisting need for LT
CKD	Unique population due to: ✓ increased prevalence of CKD in HCV ✓ high prevalence of HCV infection in patients on hemodialysis ✓ increased risk of all-cause mortality	Special attention not required. No need for caution about SOF-based regimens	None
HCV/HIV infection	Historically considered difficult- to-treat due to low SVR rates	No longer a unique population. Attention only to few interactions between DAA and anti-retroviral drugs	None
HCV/HBV infection	Historically considered at risk of developing complications (e.g., cirrhosis and HCC) compared to those with mono-infection.	Relevant risk of HBV re-activation in HBsAg positive during and after DAA therapy	Agreement among international societies regarding the best therapeutic approach
Unusual subtypes	Unknown	Emerging challenge due to the paucity of data	Future studies expected to clarify the best DAA regimen
Prior DAA failure	Unknown	Emerging challenge due to uncertainties regarding the recommended therapeutic approach	Prospective studies expected to validate international societies’ recommendations

Abbreviations: CKD, chronic kidney disease; DAA, direct-acting antivirals; HBV, hepatitis B virus; HCC hepatocellular carcinoma; HCV, hepatitis C virus, HIV, human immunodeficiency virus; LT, liver transplantation; SOF, sofosbuvir.

## Data Availability

Not applicable.
